# Quality of Life in Patients With Severe Mental Illness in a Tertiary Care Unit in Greece: The Role of Sociodemographic, Clinical Factors, and Social Support

**DOI:** 10.7759/cureus.77462

**Published:** 2025-01-15

**Authors:** Maria Dalliou, Konstantinos Bonotis

**Affiliations:** 1 Department of Social Work, University General Hospital Larissa, Larissa, GRC; 2 Faculty of Medicine School of Health Sciences, University of Thessaly, Larissa, GRC

**Keywords:** affective disorder, hospitalised patients, involuntary hospitalization, perceived social support, quality of life (qol), schizoprhrenia spectrum disorder, severe mental illness, subjective quality of life, voluntary hospitalization, who quality of life brief version (whoqol-bref)

## Abstract

Objectives

The aim of study was to evaluate the quality of life (QOL) across different diagnostic categories in inpatients with severe mental illness (SMI) and examine its associations with sociodemographic, illness-related characteristics, and perceived social support.

Methods

A cross-sectional study was conducted. A total of 280 patients, consecutively admitted, were recruited in the Adult Psychiatric Department of the University Hospital Larissa, Larisa, Greece. QOL and social support were measured using the World Health Organization Quality of Life - BREF (WHOQOL-BREF) and the Social Support Questionnaire - Short Form (SSQ-6) scales, along with sociodemographic and clinical data.

Results

The social relationships domain of the WHOQOL-BREF received the lowest score across all diagnoses. Significant differences in QOL were noted based on the type of admission, though not by diagnosis. Women reported significantly worse physical (-0.90, p=0.019), mental (-1.02, p=0.020), and overall QOL (-1.09, p=0.035) compared to men. Age was significantly associated with environmental (-0.03, p=0.016) and overall QOL (-0.05, p=0.014). Homelessness was linked to poorer QOL in social (-2.42, p=0.040) and environmental domains (-2.16, p=0.020). Urbanicity (1.09, p=0.041) and retirement (-1.48, p=0.031) were also significant factors for global QOL. Social support satisfaction had a significant positive association with the social relationship dimension (0.06, p=0.022), while the support network had a marginal effect (0.05, p=0.080). Outpatient monitoring was significantly associated with the mental health dimension (-0.94, p=0.032). Multiple regression analyses indicated that gender, marital status, age, work status, number of cohabitants, type of residence, and social support were significantly associated with global and specific QOL dimensions.

Conclusions

This study explores the QOL in inpatients with SMI, admitted both involuntarily and voluntarily, emphasizing the impact of sociodemographic, clinical factors, and perceived social support. Key predictors of QOL include female gender, homelessness, retirement, and perceived social support. Routine QOL assessments combined with gender- specific and social support interventions are essential for improving patient outcome.

## Introduction

During the previous decades, a change has been observed in the management of severe mental illness (SMI) to a more holistic approach that encompasses symptom reduction and aspects of perceived well-being [1-3]. Nowadays, it is widely recognized that the quality-of-life notion provides the best framework for evaluating treatment outcomes and offers a valuable way to include the client’s perspective in the assessment process [4-8]. Social variables related to material and social contextual factors are essential to evaluate the quality of life (QOL) of individuals with SMI [9]. In our study, SMI includes a range of disorders that require hospitalization and intensive care, such as psychotic disorders and mood disorders.

Several studies have indicated that individuals with SMI have lower subjective QOL scores compared to the general population, as measured by the World Health Organization Quality of Life - BREF (WHOQOL-BREF) questionnaire [10-14]. Factors influencing QOL are multidimensional. Recent studies using this tool have highlighted the complex interplay between sociodemographic and clinical factors and QOL [10,14-20]. Key findings revealed significant associations between age [10,21], gender [10,22-24], marital status [17], educational level [23], income [10,13], and occupation [25], with various QOL dimensions. Additionally, illness duration [21] and medication adherence [18,26] were identified as significant moderators of QOL. Surprisingly, one study reported poor QOL in schizophrenia despite improved medication adherence while it found no significant relationship between QOL and clinical factors [25]. Similarly, Çelik et al. found that patients with schizophrenia (n=74) had significantly lower scores in almost all WHOQOL-BREF dimensions (except the environmental domain) compared to those with bipolar disorder (n=124) [14].

Previous research highlighted that the availability and adequacy of social support can profoundly influence life satisfaction. This effect stems not only from the tangible benefits of support but also from its role in shaping perceptions and norms by offering a reference point for what is considered appropriate in various life domains [27]. Social support consistently emerged as a key predictor of QOL in various contexts [28-34]. For instance, larger social networks were associated with improved subjective QOL in homeless populations [35] and adults in partial hospitalization programs [36]. A cross-sectional study identified perceived social support as a significant predictor of QOL (measured with WHOQOL-BREF) in outpatients suffering from schizophrenia (n=160). Along with disorder duration, economic status, and emotional symptom severity, it explained 47.3% of the variance in QOL [28]. Moreover, perceived social support remains a critical determinant of subjective QOL in post-acute patients suffering from schizophrenia [37] and a crucial element absent from patients receiving official mental health care [32]. Many studies in quality-of-life research highlighted the need for personalized, therapeutic, and comprehensive interventions incorporating the biopsychosocial model of care [19,21,26,38].

Congruent with the above we sought to (a) assess and compare QOL among hospitalized patients (voluntary and involuntary) in a tertiary care setting, (b) compare QOL between different diagnostic categories, (c) determine environmental elements (social support), and illness-related and sociodemographic variables that are related to subjective QOL, and (d) identify which patient characteristics predict general QOL, thereby emphasizing the critical role of assessing and enhancing QOL as a core component of therapeutic interventions.

## Materials and methods

A hospital-based cross-sectional study was conducted between March 2021 and October 2023, including patients from the Adult Psychiatric Department of the University Hospital Larissa (UHL), Larisa, Greece. This study employed a cross-sectional design.

Inclusion criteria involved patients meeting the International Statistical Classification of Diseases and Related Health Problems 10th Revision (ICD-10) criteria for mood disorders, schizoaffective disorders, schizophrenia, anxiety disorders, and personality and addiction-related disorders, and those who were capable of providing written informed consent. Exclusion criteria included hospitalizations shorter than four days, presence of organic brain syndromes or severe mental impairments, refusal to participate in the study, refugee status due to language barriers and the unavailability of interpreters, inability to answer questions due to severe cognitive deficits, language barriers, medical conditions that interfered with participation, and inadequate cognitive ability as assessed by the attending psychiatrist. For patients with multiple hospitalizations, data from only one admission were included to avoid duplication.

The study protocol was approved by UHL's Scientific Board. Patients who met the inclusion criteria were invited to participate after the study's purpose and procedures were fully explained. Participants signed a consent form acknowledging their voluntary participation and the confidentiality of their data. Data were collected through individual interviews lasting 15-20 minutes, with the researcher assisting participants in completing two structured questionnaires, including the WHOQOL-BRE and Social Support Questionnaire - Short Form (SSQ-6). Clinical data were obtained by reviewing medical records.

Instruments

Sociodemographic and Clinical Measures

A form was created for the study's aims to gather the necessary data where the subsequent subsections encompassed the following.

Demographic characteristics: These included gender, age, family status, educational, occupational, economic status, medical insurance, place of residence, current living conditions, and number of cohabitants.

Clinical characteristics: Diagnosis was according to the ICD-10 Classification of Mental and Behavioral Disorders (at discharge), substance comorbidity, somatic comorbidity, duration of the disease, previous number of hospitalizations, duration of hospitalization of the present episode, medical adherence, (yes/no), and existence of outpatient psychiatric monitoring prior to admission.

Assessment of clinical characteristics: The assessment of all clinical characteristics, including medication adherence, was conducted through a two-step process: self-reported data (participants were asked to respond to relevant questions as part of the administered questionnaire) and verification through medical records (the self-reported data were cross-referenced with information from the medical records to ensure accuracy; in cases where discrepancies were found, the final status was determined based on the medical records, as they provided the most reliable and objective source of information).

Quality of life (WHOQOL-BREF)

The WHOQOL-BREF evaluates subjective QOL. In the present study, the Greek-validated version of the abbreviated version was used [39]. The WHOQOL-BREF comprises 26 items categorized into four areas: physical health, psychological health, social interactions, and environmental dimension. The tool also has a generic aspect in addition to these domains. The Greek version, including four new questions emerged in the context of cultural adaptation of the questionnaire, is shown to be a reliable and valid assessment tool, which can be used in healthy and clinical groups [39]. Four 5-point Likert answer scales (intensity, capacity, frequency, and evaluation) ranked negatively or favorably are used to create the instrument's questions. The results were evaluated on a scale from 0 to 100. The reliability of internal consistency was high for all domains of the WHOQOL-BREF. Specifically, Cronbach's α for the four domains in this study were physical 0.790, psychological 0.829, social relationships 0.730, and environment 0.733.

Social Support Questionnaire - Short Form (SSQ-6)

The quantity (network) and quality (satisfaction) of social support were measured using the 6-item self-report version of Sarason et al.’s Social Support Questionnaire (1987) [40]. The SSQ-6 consists of six items, each measuring two aspects: number of supports (SSQN) and satisfaction with support (SSQS). SSQN is calculated as the total number of people reported across all six items, divided by six to yield an average. For each item, participants listed the initials of zero to nine people to whom they can turn in different situations, with the SSQN total score ranging from 0 to 54. SSQS is calculated by averaging the satisfaction ratings (scored on a 6-point Likert scale from 1 = very dissatisfied to 6 = very satisfied) across all six items, with the SSQS total score ranging from 6 to 36. According to Uchino, the SSQ is one of the few instruments that allows a comprehensive assessment by distinguishing functional and structural aspects of social support [41]. In the present study, the Greek adapted form by Roussi and Vassilaki was used [42]. The results obtained from our study demonstrate strong reliability for both subscales: the Cronbach’s alpha for the SSQN subscale was 0.848 and that for the SSQS subscale was 0.912.

Statistical analysis

Statistical analyses were carried out using R language (Foundation for Statistical Computing, Vienna, Austria; https://www.r-project.org/). Continuous variables were expressed as mean ± standard deviation and as median with interquartile range (IQR), and categorical variables were expressed as frequencies (n) and percentages (%). Categorical data were analyzed using the chi-square test or Fisher’s exact test. Continuous variables were checked for deviation from normal distribution (Kolmogorov-Smirnoff normality test) for each comparison. Student’s t-test, ANOVA, Mann-Whitney U test, and Kruskal-Wallis test were used to assess continuous data, as appropriate. Pearson’s correlation test or Spearman’s rank correlation coefficient was used in determining the relationship between continuous variables. Multivariate analysis was performed using binary logistic regression and multiple linear regression. Finally, “all” sociodemographic and clinical variables (independent variables) were entered into multiple linear regression analysis (stepwise) or binary logistic regression (stepwise) to determine the amount of variance within the four domain scores of WHOQOL-BREF, the scores of SSQ-6, and the involuntary/voluntary admission (dependent variables) explained by them. For all the analyses, a 5% significance level was set, and p-values were two-tailed.

## Results

The research sample consisted of 280 inpatients, of whom 180 (64.2%) were admitted involuntarily and 100 (35.8%) voluntarily to the Adult Psychiatric Department of UHL. Table 1 and Table 2 show all the selected sociodemographic and clinical characteristics of the respondents.

**Table 1:** Sociodemographic characteristics of the sample IQR is presented as (Q1-Q3), where Q1 is the first quartile and Q3 is the third quartile. Percentages are based on the total sample size (N = 280). IQR, interquartile range; N (%), number of participants (percentage)

Variables	Median (IQR)/N (%)
Age (years)	50.0 (41.0-58.0)
Gender	
Male	179 (63.9%)
Female	101 (36.1%)
Marital status	
Single	164 (58.6%)
Married	60 (21.4%)
Divorced/windowed	56 (20.0%)
Number of children	0 (0.0-2.0)
Education level	
Illiterate	7 (2.5%)
Primary	55 (19.6%)
Middle	45 (16.1%)
Secondary	64 (22.9%)
Technical school	61 (21.8%)
University/postgraduate degree	47 (16.8%)
Other	1 (0.4%)
Urbanicity	
Rural	91 (32.5%)
Urban	189 (67.5%)
Residence type	
Apartment	144 (51.4%)
Detached house	125 (44.6%)
Other	11 (3.9%)
Housing condition	
Owned residence	213 (76.1%)
Rented residence	30 (10.7%)
Mortgaged residence	4 (1.4%)
Living as a guest	24 (8.6%)
Homelessness	8 (2.9%)
Other	1 (0.4%)
Work status	
Employee	62 (22.1%)
Retired (disability pension/allowance)	91 (32.5%)
Unemployed	127 (45.4%)
Monthly income	
<300€	73 (26.1%)
<600€	73 (26.1%)
<900€	27 (9.6%)
<1,200€	20 (7.1%)
>1,200€	16 (5.7%)
No income	71 (25.4%)
Medical insurance	
No	96 (34.3%)
Yes	184 65.7%)
Cohabitants (number)	1.0 (0.0-2.0)

**Table 2:** Clinical characteristics for the sample Percentages are based on the total sample size (N = 280). IQR is presented as (Q1-Q3), where Q1 is the first quartile and Q3 is the third quartile. ICD-10, International Classification of Diseases, 10th Revision; IQR, interquartile range; N (%), number of participants (percentage)

Variables	N (%)/median (IQR)
Diagnosis (ICD-10)	
F10-F19	27 (9.6%)
F20-F29	140 (50.0%)
F30-F39	94 (33.6%)
F40-F48	7 (2.5%)
F60-F69	12 (4.3%)
Illness duration	
≤1	17 (6.1%)
1-3	38 (13.6%)
4-9	58 (20.7%)
10-19	77 (27.5%)
20-29	69 (24.6%)
30-39	16 (5.7%)
≥40	5 (1.8%)
Medical adherence	
Yes	114 (40.7%)
No	166 (59.3%)
Previous admissions	
0	74 (26.4%)
1-3	106 (37.9%)
4-6	36 (12.9%)
7-9	27 (9.6%)
>10	37 (13.2%)
Substance comorbidity	
Yes	66 (23.6%)
No	214 (76.4%)
Outpatient psychiatric monitoring	
Yes	174 (62.1%)
No	106 (37.9%)
Somatic comorbidity	
Yes	60 (21.4%)
No	219 (78.2%)
Missing	1 (0.4%)
Admission duration (days)	21.0 (13.0-30.0)

Levels of QOL domain scores (WHOQOL-BREF) and type of admission

The data highlight that the legal type of admission is strongly associated with certain QOL dimensions and overall QOL. Involuntarily admitted patients reported better physical health, mental health, and overall QOL compared to voluntarily admitted patients. However, there were no notable differences in social relationships and environmental domain between the two groups (Table 3).

**Table 3:** Univariate associations between QOL domains scores (WHOQOL-BREF) and type of admission IQR is presented as (Q1-Q3), where Q1 is the first quartile and Q3 is the third quartile. Missing values are reported as the number and percentage of missing observations per variable ^a^Mann–Whitney U test IQR, interquartile range; SD, standard deviation

WHOQOL-BREF domains	Group of involuntary admitted patients (N=180)	Group of voluntary admitted patients (N=100)	Total sample of patients (N=280)	Test statistic	p-Value
Physical health domain					
Mean (SD)	14.2 (2.79)	12.0 (3.19)	13.4 (3.11)	5,654.5^a^	≤0.001^a^
Median (IQR)	14.3 (12.6-16.6)	12.0 (9.7-14.9)	13.7 (11.4-16.0)		
Missing	1 (0.6%)	0 (0%)	1 (0.4%)		
Mental health domain					
Mean (SD)	13.9 (3.03)	11.3 (3.83)	13.0 (3.55)	5,511^a^	≤0.001^a^
Median (IQR)	14.0 (12.0-16.0)	12.0 (8.7-14.7)	13.3 (10.7-15.3)		
Missing	1 (0.6%)	0 (0%)	1 (0.4%)		
Social relationships domain					
Mean (SD)	11.9 (3.85)	11.4 (3.73)	11.7 (3.81)	8,358^a^	0.320^a^
Median (IQR)	12.0 (9.3-14.7)	12.0 (8.0-14.7)	12.0 (8.0-14.7)		
Missing	2 (1.1%)	0 (0%)	2 (0.7%)		
Environmental domain					
Mean (SD)	13.2 (2.47)	12.6 (2.75)	13.0 (2.59)	7,992^a^	0.120^a^
Median (IQR)	13.5 (11.5-15.0)	13.0 (10.5-14.5)	13.0 (11.0-15.0)		
Missing	1 (0.6%)	0 (0%)	1 (0.4%)		
Overall quality of life					
Mean (SD)	13.1 (3.92)	11.1 (4.35)	12.4 (4.18)	6,725.5^a^	≤0.001^a^
Median (IQR)	14.0 (10.0-16.0)	12.0 (8.0-14.0)	12.0 (10.0-16.0)		
Missing	1 (0.6%)	0 (0%)	1 (0.4%)		

QOL domain scores (WHOQOL-BREF) in different diagnostic groups (ICD-10) among inpatients with severe mental illness

Across all diagnostic categories, the social relationships domain had the lowest mean and median scores compared to other QOL dimensions. Specifically, when comparing patients with schizophrenia spectrum disorders (F20-F29) and affective disorders (F30-F39), the following trends were observed: (a) patients with schizophrenia spectrum disorders demonstrated higher scores in the physical health domain, (b) patients with affective disorders scored higher in the mental health, social relationships, and environmental domains, (c) no significant differences in overall QOL were found between these groups. Despite these trends, all p-values indicated no statistically significant associations between diagnostic categories and QOL subdomains or overall QOL. Detailed descriptive statistics and p-values for each domain are provided in Table 4.

**Table 4:** Relationship between diagnosis (ICD-10) and quality of life domains scores (WHOQOL-BREF) among inpatients with severe mental illness IQR is presented as (Q1-Q3), where Q1 is the first quartile and Q3 is the third quartile. Missing values are reported as the number and percentage of missing observations per variable ^a^The test statistic is adjusted for ties. ^H^H-statistic. ^K-W^Kruskal-Wallis test IQR, interquartile range; SD, standard deviation

	F10-F19 (N=27)	F20-F29 (N=140)	F30-F39 (N=94)	F40-F48 (N=7)	F60-F69 (N=12)	Total (N=280)	Test statistic	p-Value
Physical health domain								
Mean (SD)	13.3 (2.82)	13.5 (3.05)	13.2 (3.29)	12.6 (3.41)	14.2 (3.14)	13.4 (3.11)	2.373^Η^	0.668^a, K-W^
Median (IQR)	13.1 (11.7-14.9)	13.7 (12.0-16.0)	13.1 (9.7-14.9)	13.7 (10.9-14.3)	14.9 (11.7-15.3)	13.7 (10.3-15.4)		
Missing	0 (0%)	1 (0.7%)	0 (0%)	0 (0%)	0 (0%)	1 (0.4%)		
Mental health domain								
Mean (SD)	13.2 (3.09)	12.8 (3.36)	13.1 (3.94)	12.0 (3.81)	13.7 (3.58)	13.0 (3.55)	1.864^Η^	0.761^a, K-W^
Median (IQR)	14.0 (13.0-14.7)	13.3 (11.2-16.0)	14.0 (10.7-15.3)	12.7 (11.2-14.5)	13.7 (12.5-15.3)	13.3 (11.3-15.3)		
Missing	0 (0%)	1 (0.7%)	0 (0%)	0 (0%)	0 (0%)	1 (0.4%)		
Social relationships domain								
Mean (SD)	11.7 (3.63)	11.4 (3.81)	12.2 (3.87)	10.3 (2.85)	13.2 (3.96)	11.7 (3.81)	5.244^Η^	0.263^a, K-W^
Median (IQR)	10.7 (8.7-13.3)	11.3 (10.0-16.0)	12.7 (8.0-15.0)	10.7 (11.0-12.7)	13.3 (10.0-16.3)	12.0 (9.3-16.0)		
Missing	0 (0%)	2 (1.4%)	0 (0%)	0 (0%)	0 (0%)	2 (0.7%)		
Environmental domain								
Mean (SD)	13.1 (2.22)	12.9 (2.66)	12.9 (2.65)	13.2 (1.50)	13.4 (2.80)	13.0 (2.59)	0.217^Η^	0.995^a, K-W^
Median (IQR)	13.5 (13.5-14.8)	13.0 (12.0-14.5)	13.3 (11.0-15.1)	13.0 (13.0-14.0)	13.0 (12.2-16.1)	13.0 (11.5-15.0)		
Missing	0 (0%)	1 (0.7%)	0 (0%)	0 (0%)	0 (0%)	1 (0.4%)		
Overall quality of life								
Mean (SD)	13.0 (3.11)	12.3 (4.09)	12.3 (4.64)	12.0 (4.62)	13.0 (3.67)	12.4 (4.18)	0.482^Η^	0.975^a, K-W^
Median (IQR)	14.0 (13.0-16.0)	12.0 (12.0-16.0)	12.0 (8.0-14.5)	12.0 (10.5-14.5)	12.0 (12.0-14.5)	12.0 (11.5-16.0)		
Missing	0 (0%)	1 (0.7%)	0 (0%)	0 (0%)	0 (0%)	1 (0.4%)		

Associations of QOL domains scores (WHOQOL-BREF) with sociodemographic, clinical characteristics, and social support (SSQ-6) among inpatients with severe mental illness: results from univariate and multivariate analyses

Univariate analyses highlighted multiple associations between QOL domains and patient characteristics (Table 5). Women reported significantly lower scores across physical domain, psychological domain, and overall QOL compared to men. Married individuals showed slightly worse physical health outcomes than singles, while a marginal trend suggested better scores for those with one to three previous hospitalizations compared to none. In the psychological domain, outpatient psychiatric care and female gender were associated with lower scores. For social relationships, living in detached houses or experiencing homelessness correlated with poorer satisfaction. Similarly, patients with more than 10 previous admissions reported reduced satisfaction, while stronger perceived social support contributed to better social relationships. Environmental health was negatively associated with female gender, older age, and homelessness, all of which correlated with lower satisfaction. Lastly, overall QOL was significantly influenced by age, gender, urbanicity, and retirement status. Divorced or widowed individuals exhibited a tendency toward lower QOL, although this finding was marginal.

**Table 5:** Univariate analyses of sociodemographic, clinical factors, and social support (SSQ-6) associated with QOL domains scores (WHOQOL-BREF) among inpatients with severe mental illness Values represent regression coefficients (95% confidence intervals) and p-values from univariate regression analyses. Ref: group used for comparison in the univariable analysis. *Values represent the score range of the questionnaire responses.

Variables	Physical health domain		Mental health domain		Social relationships domain		Environmental domain		Overall quality of life	
	t-value	Coefficient (univariable)	t-value	Coefficient (univariable)	t-value	Coefficient (univariable)	t-value	Coefficient (univariable)	t-value	Coefficient (univariable)
Age (19-90 years)	-1.142	-0.02 (-0.04 to 0.01, p=0.254)	-0.926	-0.01 (-0.05 to 0.02, p=0.355)	-1.854	-0.03 (-0.07 to 0.00, p=0.065)	-2.425	-0.03 (-0.05 to -0.01, p=0.016)	-2.485	-0.05 (-0.08 to -0.01, p=0.014)
Gender										
Male		Ref.		Ref.		Ref.		Ref.		Ref.
Female	-2.352	-0.90 (-1.66 to -0.15, p=0.019)	-2.342	-1.02 (-1.89 to -0.16, p=0.020)	0.270	0.13 (-0.81 to 1.06, p=0.786)	-1.713	-0.55 (-1.18 to 0.08, p=0.088)	-2.117	-1.09 (-2.11 to -0.08, p=0.035)
Marital status										
Not married		Ref.		Ref.		Ref.		Ref.		Ref.
Married	-1.909	-0.89 (-1.81 to 0.03, p=0.057)	-0.391	-0.21 (-1.27 to 0.85, p=0.696)	-0.312	-0.18 (-1.31 to 0.95, p=0.758)	0.026	0.01 (-0.76 to 0.78, p=0.980)	-1.060	-0.67 (-1.90 to 0.57, p=0.290)
Divorced/windowed	-0.702	-0.34 (-1.28 to 0.61, p=0.483)	0.005	0.00 (-1.08 to 1.09, p=0.996)	1.128	0.67 (-0.50 to 1.83, p=0.260)	-0.835	-0.33 (-1.12 to 0.45, p=0.404)	-1.910	-1.23 (-2.50 to 0.04, p=0.057)
Urbanicity										
Rural		Ref.		Ref.		Ref.		Ref.		Ref.
Urban	-0.326	-0.13 (-0.91 to 0.65, p=0.744)	-0.150	-0.07 (-0.96 to 0.82, p=0.881)	0.481	0.23 (-0.73 to 1.19, p=0.632)	1.263	0.42 (-0.23 to 1.06, p=0.208)	2.054	1.09 (0.05 to 2.13, p=0.041)
Type of residence										
Apartment		Ref.		Ref.		Ref.		Ref.		Ref.
Detached house	-0.519	-0.20 (-0.94 to 0.55, p=0.604)	-0.904	-0.39 (-1.24 to 0.46, p=0.367)	-3.147	-1.44 (-2.34 to -0.54, p=0.002)	-0.530	-0.17 (-0.78 to 0.45, p=0.596)	-0.939	-0.48 (-1.48 to 0.52, p=0.348)
Other	-1.387	-1.35 (-3.26 to 0.57, p=0.167)	-1.529	-1.69 (-3.87 to 0.49, p=0.127)	-2.069	-2.42 (-4.72 to -0.12, p=0.040)	-2.363	-1.90 (-3.48 to -0.32, p=0.019)	-1.487	-1.94 (-4.51 to 0.63, p=0.138)
Housing conditions										
Owned residence		Ref.		Ref.		Ref.		Ref.		Ref.
Rented residence	-0.809	-0.49 (-1.68 to 0.70, p=0.419)	-0.719	-0.49 (-1.85 to 0.86, p=0.473)	0.250	0.19 (-1.27 to 1.64, p=0.799)	-1.087	-0.54 (-1.53 to 0.44, p=0.278)	-0.436	-0.35 (-1.95 to 1.24, p=0.663)
Mortgaged residence	-0.476	-0.74 (-3.82 to 2.33, p=0.634)	-0.390	-0.70 (-4.20 to 2.81, p=0.697)	-1.147	-2.19 (-5.95 to 1.58, p=0.253)	-1.283	-1.66 (-4.21 to 0.89, p=0.201)	-0.439	-0.92 (-5.05 to 3.21, p=0.661)
Living as a guest	1.200	0.80 (-0.51 to 2.11, p=0.231)	0.878	0.67 (-0.83 to 2.16, p=0.381)	-0.265	-0.22 (-1.82 to 1.39, p=0.792)	-0.629	-0.35 (-1.43 to 0.74, p=0.530)	1.300	1.16 (-0.60 to 2.92, p=0.195)
Homelessness	-1.051	-1.17 (-3.37 to 1.02, p=0.294)	-0.809	-1.03 (-3.53 to 1.47, p=0.419)	-1.485	-2.02 (-4.71 to 0.66, p=0.140)	-2.340	-2.16 (-3.98 to -0.34, p=0.020)	-1.617	-2.42 (-5.37 to 0.53, p=0.107)
Work status										
Employee		Ref.		Ref.		Ref.		Ref.		Ref.
Retired (disability pension/allowance)	-0.829	-0.42 (-1.43 to 0.58, p=0.408)	0.045	0.03 (-1.13 to 1.18, p=0.964)	-1.889	-1.18 (-2.41 to 0.05, p=0.060)	-0.168	-0.07 (-0.91 to 0.77, p=0.867)	-2.163	-1.48 (-2.83 to -0.13, p=0.031)
Unemployed	-0.697	-0.34 (-1.29 to 0.61, p=0.486)	-0.217	-0.12 (-1.20 to 0.96, p=0.828)	-1.762	-1.04 (-2.19 to 0.12, p=0.079)	0.492	0.20 (-0.59 to 0.99, p=0.623)	-1.151	-0.74 (-2.01 to 0.53, p=0.251)
SSQ-6 scale (network) (0.0-54.0)*	-0.955	-0.02 (-0.07 to 0.03, p=0.340)	-0.386	-0.01 (-0.07 to 0.05, p=0.700)	1.759	0.05 (-0.01 to 0.12, p=0.080)	-0.419	-0.01 (-0.05 to 0.03, p=0.675)	-1.382	-0.05 (-0.11 to 0.02, p=0.168)
SSQ-6 scale (satisfaction) (0.0-36.0)*	1.212	0.03 (-0.02 to 0.07, p=0.226)	0.422	0.01 (-0.04 to 0.06, p=0.674)	2.309	0.06 (0.01 to 0.11, p=0.022)	1.841	0.03 (-0.00 to 0.07, p=0.067)	0.584	0.02 (-0.04 to 0.07, p=0.560)
Outpatient psychiatric monitoring										
No		Ref.		Ref.		Ref.		Ref.		Ref.
Yes	-0.491	-0.19 (-0.94 to 0.57, p=0.624)	-2.161	-0.94 (-1.79 to -0.08, p=0.032)	-0.353	-0.17 (-1.09 to 0.76, p=0.723)	0.055	0.02 (-0.61 to 0.65, p=0.956)	-1.362	-0.70 (-1.71 to 0.31, p=0.174)
Medical adherence										
No		Ref.		Ref.		Ref.		Ref.		Ref.
Yes	-1.487	-0.56 (-1.30 to 0.18, p=0.138)	-1.711	-0.73 (-1.58 to 0.11, p=0.088)	-0.269	-0.12 (-1.04 to 0.79, p=0.790)	-0.085	-0.03 (-0.65 to 0.59, p=0.932)	-0.202	-0.10 (-1.10 to 0.90, p=0.840)
Number of hospitalizations										
0		Ref.		Ref.		Ref.		Ref.		Ref.
1-3	1.782	0.84 (-0.09 to 1.76, p=0.076)	-0.020	-0.01 (-1.07 to 1.05, p=0.984)	-1.585	-0.91 (-2.03 to 0.22, p=0.115)	-1.203	-0.47 (-1.24 to 0.30, p=0.230)	-0.484	-0.31 (-1.55 to 0.94, p=0.628)
4-6	0.470	0.30 (-0.94 to 1.53, p=0.639)	-0.209	-0.15 (-1.57 to 1.27, p=0.834)	-0.885	-0.68 (-2.21 to 0.85, p=0.381)	-1.410	-0.74 (-1.77 to 0.29, p=0.160)	-0.444	-0.38 (-2.05 to 1.29, p=0.657)
7-9	0.217	0.15 (-1.22 to 1.52, p=0.828)	-0.838	-0.67 (-2.24 to 0.90, p=0.403)	-1.617	-1.37 (-3.05 to 0.30, p=0.108)	-1.626	-0.94 (-2.08 to 0.20, p=0.105)	-0.855	-0.80 (-2.65 to 1.05, p=0.394)
>10	0.169	0.11 (-1.12 to 1.33, p=0.866)	-0.635	-0.45 (-1.86 to 0.95, p=0.526)	-2.118	-1.61 (-3.11 to -0.11, p=0.035)	-0.775	-0.40 (-1.42 to 0.62, p=0.439)	-0.845	-0.71 (-2.37 to 0.94, p=0.399)
Involuntary type of admission										
No		Ref.		Ref.		Ref.		Ref.		Ref.
Yes	-5.866	1.27 (1.17 to 1.39, p<0.001)	-6.051	1.24 (1.15 to 1.34, p<0.001)	-1.073	1.04 (0.97 to 1.11, p=0.285)	-1.795	1.09 (0.99 to 1.20, p=0.075)	-3.848	2.47 (1.25 to 3.70, p<0.001)

Multiple regression analyses

The multiple regression analyses (Table 6) identified distinct predictors for each QOL domain. In the physical health domain, female gender, marital status, and larger social support networks were associated with lower scores, while living with more cohabitants was linked to better outcomes. Satisfaction with social support showed a marginally positive association with physical health. Mental health was positively influenced by the number of children but negatively associated with female gender. Similarly, outpatient psychiatric monitoring showed a marginally negative trend. Social relationships domain was significantly influenced by residence type and satisfaction with social support, with individuals in detached houses or perceiving lower social support satisfaction experiencing poorer outcomes. In the environmental domain, older age, type of residence (other - primarily referring to homelessness), and larger social support networks were associated with poorer outcomes, while satisfaction with social support improved scores, suggesting a complex relationship. Finally, for overall QOL, retirement and larger social networks emerged as significant predictors of lower scores, while urban residency appeared to support higher QOL. These findings highlight the multifaceted nature of QOL determinants and underscore the importance of addressing social and clinical factors in this population.

**Table 6:** Multiple linear regression analyses (backward stepwise) of sociodemographic, clinical, and social support (SSQ-6) predictors for individual domains and overall quality of life (WHOQOL-BREF) among inpatients with severe mental illness Values represent regression coefficients (95% confidence intervals) and p-values from multiple regression analysis (backward stepwise) DF, degrees of freedom

Dependent Variable	Independent Variable	t-value	Coefficient (95% CI)	P-value	Model			
					Multiple R-squared	Adjusted R-squared	F-statistic	p-value
Physical health domain	Female gender	-2.068	-0.82 (-1.60 to -0.04)	0.040	0.093	0.056	2.504 on 11 and 268 DF	0.005
	Marital status: married vs single	-2.005	-0.96 (-1.91 to -0.02)	0.046				
	Marital status: divorced /widowed vs single	-0.005	-	0.996				
	Number of cohabitants	2.885	0.39 (0.12 to 0.65)	0.004				
	Housing conditions: rented residence vs owned residence	-0.535	-0.32 (-1.48 to 0.85)	0.593				
	Housing conditions: mortgaged residence vs owned residence	-0.818	-1.26 (-4.31 to 1.78)	0.414				
	Housing conditions: living as a guest vs owned residence	1.163	0.77 (-0.53 to 2.06)	0.246				
	Housing conditions: homelessness vs owned residence	-0.57	-0.65 (-2.88 to 1.59)	0.569				
	Social support (network)	-2.113	-0.06 (-0.12 to -0.001)	0.036				
	Social support (satisfaction)	1.85	0.05 (-0.001 to 0.09)	0.065				
Mental health domain	Age	-1.43	-0.02 (-0.06 to 0.01)	0.154	0.056	0.039	3.259 on 5 and 274 DF	0.007
	Female gender	-2.308	-1.01 (-1.88 to -0.15)	0.022				
	Number of children	2.129	0.42 (0.03 to 0.81)	0.034				
	Medical insurance (yes)	-1.153	-0.70 (-1.60 to 0.20)	0.127				
	Outpatient monitoring	-1.833	-0.80 (-1.65 to 0.06)	0.068				
Social relationships domain	Residence type: detached house vs apartment)	-3.094	-1.41 (-2.31 to -0.51)	0.002	0.056	0.046	5.488 on 3 and 276 DF	0.001
	Residence type: other vs apartment)	-1.73	-2.04 (-4.36 to 0.28)	0.085				
	Social support (satisfaction)	2.025	0.05 (0.01 to 0.10)	0.044				
Environmental domain	Age	-2.223	-0.03 (-0.05 to -0.001)	0.027	0.067	0.046	3.265 on 6 and 273 DF	0.004
	Female gender	-1.574	-0.50 (-1.12 to 0.12)	0.117				
	Residence type: detached house vs apartment	-0.435	-0.14 (-0.75 to 0.48)	0.664				
	Residence type: other vs apartment	-2.077	-1.67 (-3.26 to -0.09)	0.039				
	Social support (network)	-2.137	-0.05 (-0.10 to -0.001)	0.034				
	Social support (satisfaction)	2.217	0.04 (0.01 to 0.08)	0.027				
Overall quality of life	Work status: retired vs employee	-2.238	-1.59 (-2.99 to -0.19)	0.026	0.074	0.054	3.644 on 6 and 273 DF	0.002
	Work status: unemployed vs employee	-1.567	-1.02 (-2.30 to 0.26)	0.118				
	Social support (network)	-2.141	-0.08 (-0.15 to -0.01)	0.033				
	Age	-1.775	-0.04 (-0.08 to 0.001)	0.077				
	Female gender	-1.891	-0.98 (-1.99 to 0.04)	0.060				
	Urbanicity	1.868	1.00 (-0.05 to 2.06)	0.063				

## Discussion

To the best of our knowledge, this is the first hospitalized-based study conducted in Greece within acute care setting that investigates QOL across different diagnostic categories while considering sociodemographic factors, illness-related characteristics, and perceived social support. The study’s findings highlight complex associations between QOL dimensions, type of hospitalization, demographic and clinical variables, and perceived social support.

Among the four QOL dimensions, the social relationships domain consistently scored the lowest across all respondents, especially among involuntarily hospitalized patients. While this finding was not statistically significant, it aligns with studies from other countries, which reported similar results in outpatient populations with schizophrenia [18,25,43,44]. This persistent low rating may reflect the impact of SMI on social functioning and the effects of stigma, which contribute to social isolation. Addressing stigma and strengthening social networks through targeted interventions could improve this dimension of QOL.

Interestingly, while the overall QOL scores were similar for patients with schizophrenia spectrum and affective disorders, those with schizophrenia spectrum disorders reported lower scores in the mental health, social relationships, and environmental domains. This finding contrasts with the "reality distortion fallacy," which suggests that patients with psychotic disorders tend to overestimate environmental conditions [9], as well as with another study reporting better QOL in this population [45]. These differences may stem from the chronicity and severity of symptoms in schizophrenia spectrum disorders, which heighten awareness of social and environmental difficulties. Cultural factors, such as the importance of family and community ties in Greece, might further amplify perceived deficits in social relationships. These findings highlight the need for targeted interventions to address social isolation, cognitive difficulties, and environmental challenges specific to individuals with schizophrenia spectrum disorders.

A statistically significant difference in QOL was observed between involuntarily and voluntarily hospitalized patients, particularly in the physical and psychological health domains and overall QOL. This finding aligns with previous research [6], although the proportion of involuntary admissions in our sample was notably higher. Poor adherence to treatment before admission, likely linked to reduced illness insight, may partially explain this disparity as also confirmed in another study [46].

Another notable finding was the lack of association between QOL and psychiatric diagnosis, supporting another international study [25] while contrasting others [6,14]. This emphasizes the need to consider factors beyond diagnosis when assessing QOL in patients with SMI. Previous readmissions had a negative impact on the social relationship domain, echoing findings from another study [6]. Frequent admissions likely disrupt social ties, emphasizing the need for continuity of care and community-based interventions to reduce hospital readmissions. Outpatient psychiatric follow-up was negatively associated with mental health self-perception, potentially reflecting higher levels of insight and increased awareness of illness burden. Medication adherence, however, did not significantly affect QOL, contrasting with findings from other studies [18,26].

Demographic factors also influenced QOL. Age negatively correlated with overall QOL and the environmental domain, consistent with findings from China [26] but contrasting with another study showing positive correlations [21]. Female gender emerged as an independent risk factor for reduced QOL in physical and mental health domains, echoing international evidence highlighting gender disparities [6,38,47,48]. Homelessness emerged as a significant barrier to social and environmental QOL, underscoring the importance of housing stability for mental health recovery [49]. Patients receiving a pension or allowance reported lower levels of QOL compared to employed individuals, a finding consistent with international studies [6,25,50-52]. This discrepancy may be attributed to challenges associated with disability, limited social networks, and reduced opportunities for social engagement. Addressing these issues through targeted vocational rehabilitation programs, social inclusion initiatives, and mental health support services could help mitigate the impact on QOL in this group.

Social support emerged as a pivotal factor influencing QOL. Larger networks were paradoxically associated with lower physical, environmental, and global QOL, possibly due to caregiver burden and stigma. This finding is in contrast to the result of a longitudinal study in which the larger network showed better overall subjective satisfaction with QOL [53]. In contrast, satisfaction with social support positively influenced multiple QOL dimensions, similarly confirmed in many other studies [28-34]. Interventions enhancing the quality of social support could mitigate these effects and improve overall QOL. Research has shown that treatments targeted at these crucial aspects of social support can considerably improve QOL of patients with SMI [27,28].

Limitations and strengths

The study has a number of limitations that must be taken into account in the interpretation of the results. First, the cross-sectional design precludes causal inferences. Another limitation of the current study is that QOL was only evaluated at one time. Longitudinal assessment and monitoring of QOL would be interesting. Third, the study relied on self-report measurements, and thus it is unclear how much the responses of the individuals were influenced by social desirability. Furthermore, patients were not evaluated for other aspects related to QOL, such as insight [54,55], intensity of symptoms [56], general functioning [43,57], stigma [58], and family functioning [59]. These parameters should be taken into account for future research.

However, compared to previous studies on QOL, the present study has notable strengths including a large sample size, as well as the inclusion of diverse factors such as type of hospitalization, different diagnoses, and perceived social support, and reports results that are very applicable to clinical practice and patient care. There is scientific originality by integrating environmental and subjective factors in relation to QOL determinants in patients with SMI and highlighting areas for targeted interventions.

## Conclusions

This study provides valuable insights into the QOL among inpatients with SMI, admitted both involuntarily and voluntarily. It highlights significant associations between QOL dimensions and sociodemographic factors, clinical factors, and perceived social support. Given the critical role of general hospitals in community psychiatry, integrating QOL assessment into routine care is essential. Sociodemographic characteristics, such as female gender, and retirement, alongside perceived social support, emerged as key predictors of QOL. To enhance QOL, specific care plans prior to discharge should focus on addressing female vulnerability, improving social support networks, and mitigating the stigma associated with SMI. Furthermore, targeted interventions aimed at strengthening community-based mental health services and fostering vocational rehabilitation can play a crucial role in improving outcomes for this population.

## References

[1] de Almeida Mello J, Luo H, Hirdes A (2021). An International pilot study of self-reported quality of life in outpatient and inpatient mental health settings. Front Psychiatry.

[2] Connell J, Brazier J, O'Cathain A, Lloyd-Jones M, Paisley S (2012). Quality of life of people with mental health problems: a synthesis of qualitative research. Health Qual Life Outcomes.

[3] Juvva S, Newhill CE (2011). Rehabilitation contexts: a holistic approach. J Hum Behav Soc Environ.

[4] Barry MM, Zissi A (1997). Quality of life as an outcome measure in evaluating mental health services: a review of the empirical evidence. Soc Psychiatry Psychiatr Epidemiol.

[5] Orley J, Saxena S, Herrman H (1998). Quality of life and mental illness. Reflections from the perspective of the WHOQOL. Br J Psychiatry.

[6] Hodgson ZG, Pattison C, Bostock L (2007). The influence of socio-demographic and illness variables on quality of life in acute psychiatric inpatients. Clin Psychol Psychother.

[7] Baker F, Intagliata J (1982). Quality of life in the evaluation of community support systems. Eval Program Plann.

[8] Voruganti L, Heslegrave R, Awad AG, Seeman MV (1998). Quality of life measurement in schizophrenia: reconciling the quest for subjectivity with the question of reliability. Psychol Med.

[9] Katsching H (1997). How useful is the concept of quality of life in psychiatry?. Curr Opin Psychiatry.

[10] Dong M, Lu L, Zhang L (2019). Quality of life in schizophrenia: a meta-analysis of comparative studies. Psychiatr Q.

[11] Medici CR, Vestergaard CH, Hjorth P, Hansen MV, Shanmuganathan JW, Viuff AG, Munk-Jørgensen P (2016). Quality of life and clinical characteristics in a nonselected sample of patients with schizophrenia. Int J Soc Psychiatry.

[12] Brieger P, Röttig S, Marneros A (2004). [Quality of life in unipolar depressive and bipolar affective patients]. Psychiatr Prax.

[13] Islam MK, Islam MS, Kibria SM, Rahman MM, Algin S, Mullick MS (2020). Quality of life among patients with bipolar disorder. Mymensingh Med J.

[14] Çelik HE, Ceylan D, Bağcı B, Akdede BB, Alptekin K, Özerdem A (2022). Quality of life of individuals with bipolar disorder and schizophrenia. Noro Psikiyatr Ars.

[15] Olusina AK, Ohaeri JU (2003). Subjective quality of life of recently discharged Nigerian psychiatric patients. Soc Psychiatry Psychiatr Epidemiol.

[16] Doğan S, Sabanciogullari S (2003). The effects of patient education in lithium therapy on quality of life and compliance. Arch Psychiatr Nurs.

[17] Elegbede VI, Obadeji A, Adebowale TO, Oluwole LO (2019). Comparative assessment of quality of life of patients with schizophrenia attending a community psychiatric centre and a psychiatric hospital. Ghana Med J.

[18] Desalegn D, Girma S, Abdeta T (2020). Quality of life and its association with psychiatric symptoms and socio-demographic characteristics among people with schizophrenia: a hospital-based cross-sectional study. PLoS One.

[19] Cotrena C, Branco LD, Shansis FM, Fonseca RP (2020). Predictors of quality of life in bipolar disorder: a path analytical study. Psychiatry Res.

[20] Nunes KG, da Rocha NS (2022). Resilience in severe mental disorders: correlations to clinical measures and quality of life in hospitalized patients with major depression, bipolar disorder, and schizophrenia. Qual Life Res.

[21] Shumye S, Amare T, Derajew H, Endris M, Molla W, Mengistu N (2021). Perceived quality of life and associated factors among patients with severe mental illness in Ethiopia: a cross-sectional study. BMC Psychol.

[22] Mahmud MHS, Yeasmin B, Mandal S (2015). Quality of life of schizophrenic patients in a tertiary care hospital in Bangladesh. Bang J Psychiatry.

[23] Peng X, Wang S, Bi J (2021). Gender differences in socio-demographics, clinical characteristic and quality of life in patients with schizophrenia: a community-based study in Shenzhen. Asia Pac Psychiatry.

[24] Shafie S, Samari E, Jeyagurunathan A, Abdin E, Chang S, Chong SA, Subramaniam M (2021). Gender difference in quality of life (QoL) among outpatients with schizophrenia in a tertiary care setting. BMC Psychiatry.

[25] Solanki RK, Singh P, Midha A, Chugh K (2008). Schizophrenia: impact on quality of life. Indian J Psychiatry.

[26] He XY, Migliorini C, Huang ZH (2022). Quality of life in patients with schizophrenia: a 2-year cohort study in primary mental health care in rural China. Front Public Health.

[27] Baker F, Jodrey D, Intagliata J (1992). Social support and quality of life of community support clients. Community Ment Health J.

[28] Hamaideh S, Al-Magaireh D, Abu-Farsakh B, Al-Omari H (2014). Quality of life, social support, and severity of psychiatric symptoms in Jordanian patients with schizophrenia. J Psychiatr Ment Health Nurs.

[29] Fleury MJ, Grenier G, Bamvita JM, Tremblay J, Schmitz N, Caron J (2013). Predictors of quality of life in a longitudinal study of users with severe mental disorders. Health Qual Life Outcomes.

[30] Yasien S, Alvi T, Moghal F (2013). Does perceived social support predict quality of life in psychiatric patients?. Asian J Soc Sci Humanit.

[31] Ritsner M, Modai I, Endicott J (2000). Differences in quality of life domains and psychopathologic and psychosocial factors in psychiatric patients. J Clin Psychiatry.

[32] Munikanan T, Midin M, Daud TI (2017). Association of social support and quality of life among people with schizophrenia receiving community psychiatric service: a cross-sectional study. Compr Psychiatry.

[33] Roe D, Mashiach-Eizenberg M, Lysaker PH (2011). The relation between objective and subjective domains of recovery among persons with schizophrenia-related disorders. Schizophr Res.

[34] Caron J, Lecomte Y, Stip E, Renaud S (2005). Predictors of quality of life in schizophrenia. Community Ment Health J.

[35] Lam JA, Rosenheck RA (2000). Correlates of improvement in quality of life among homeless persons with serious mental illness. Psychiatr Serv.

[36] Corrigan PW, Buican B (1995). The construct validity of subjective quality of life for the severely mentally ill. J Nerv Ment Dis.

[37] Bechdolf A, Klosterkötter J, Hambrecht M, Knost B, Kuntermann C, Schiller S, Pukrop R (2003). Determinants of subjective quality of life in post acute patients with schizophrenia. Eur Arch Psychiatry Clin Neurosci.

[38] Hofer A, Baumgartner S, Edlinger M (2005). Patient outcomes in schizophrenia I: correlates with sociodemographic variables, psychopathology, and side effects. Eur Psychiatry.

[39] Ginieri-Coccossis M, Triantafillou E, Tomaras V, Soldatos C, Mavreas V, Christodoulou G (2012). Psychometric properties of WHOQOL-BREF in clinical and health Greek populations: incorporating new culture-relevant items. Psychiatriki.

[40] Sarason IG, Sarason BR, Shearin EN (1987). A brief measure of social support: practical and theoretical implications. J Soc Pers Relat.

[41] Uchino BN (2004). Social Support & Physical Health Understanding the Health Consequences of Relationships. https://books.google.gr/books?hl=en&lr=&id=VxUgC6S255wC&oi=fnd&pg=PP5&dq=Social+Support+%26+Physical+Health+Understanding+the+Health+Consequences+of+Relationships&ots=6DxjGegjck&sig=8jxbzHK2cDLyR3i1Ayvg2xny0mg&redir_esc=y#v=onepage&q=Social%20Support%20%26%20Physical%20Health%20Understanding%20the%20Health%20Consequences%20of%20Relationships&f=false.

[42] Roussi P, Vassilaki E (2001). The applicability of the multiaxial model of coping to a Greek population. Anxiety Stress Coping.

[43] Galuppi A, Turola MC, Nanni MG, Mazzoni P, Grassi L (2010). Schizophrenia and quality of life: how important are symptoms and functioning?. Int J Ment Health Syst.

[44] Guedes de Pinho LM, Pereira AM, Chaves CM (2018). Quality of life in schizophrenic patients: the influence of sociodemographic and clinical characteristics and satisfaction with social support. Trends Psychiatry Psychother.

[45] Ruggeri M, Gater R, Bisoffi G, Barbui C, Tansella M (2002). Determinants of subjective quality of life in patients attending community-based mental health services. The South-Verona Outcome Project 5. Acta Psychiatr Scand.

[46] Xiang YT, Wang Y, Wang CY (2012). Association of insight with sociodemographic and clinical factors, quality of life, and cognition in Chinese patients with schizophrenia. Compr Psychiatry.

[47] Rudolf H, Priebe S (1999). Subjective quality of life in female in-patients with depression: a longitudinal study. Int J Soc Psychiatry.

[48] (2021). Promoting mental health: concepts, emerging evidence, practice. https://www.who.int/publications/i/item/9241562943.

[49] Rae BE, Rees S (2015). The perceptions of homeless people regarding their healthcare needs and experiences of receiving health care. J Adv Nurs.

[50] Skantze K, Malm U, Dencker SJ, May PR, Corrigan P (1992). Comparison of quality of life with standard of living in schizophrenic out-patients. Br J Psychiatry.

[51] Rüesch P, Graf J, Meyer PC, Rössler W, Hell D (2004). Occupation, social support and quality of life in persons with schizophrenic or affective disorders. Soc Psychiatry Psychiatr Epidemiol.

[52] Adelufosi AO, Ogunwale A, Abayomi O, Mosanya JT (2013). Socio-demographic and clinical correlates of subjective quality of life among Nigerian outpatients with schizophrenia. Psychiatry Res.

[53] Cechnicki A, Wojciechowska A, Valdez M (2008). The social network and the quality of life of people suffering from schizophrenia seven years after the first hospitalisation. Arch Psychiatry Psychother.

[54] Sagayadevan V, Jeyagurunathan A, Lau YW (2019). Cognitive insight and quality of life among psychiatric outpatients. BMC Psychiatry.

[55] Ramadan ES, El Dod WA (2010). Relation between insight and quality of life in patients with schizophrenia: role of internalized stigma and depression. Curr Psychiatry.

[56] Tong Chien W, Thompson DR, Fong Leung S, Bressington D (2022). Quality of life, symptom severity and level of functioning in people with severe mental illness ready for hospital discharge. J Psychiatr Ment Health Nurs.

[57] Nevarez-Flores AG, Sanderson K, Breslin M, Carr VJ, Morgan VA, Neil AL (2019). Systematic review of global functioning and quality of life in people with psychotic disorders. Epidemiol Psychiatr Sci.

[58] Cheng CM, Chang CC, Wang JD, Chang KC, Ting SY, Lin CY (2019). Negative impacts of self-stigma on the quality of life of patients in methadone maintenance treatment: the mediated roles of psychological distress and social functioning. Int J Environ Res Public Health.

[59] Caqueo Urízar A, Lemos Giráldez S (2008). [Quality of life and family functioning in schizophrenia patients]. Psicothema.

